# Epigenetic Silencing of Spermatocyte-Specific and Neuronal Genes by SUMO Modification of the Transcription Factor Sp3

**DOI:** 10.1371/journal.pgen.1001203

**Published:** 2010-11-11

**Authors:** Bastian Stielow, Imme Krüger, Rolf Diezko, Florian Finkernagel, Nynke Gillemans, John Kong-a-San, Sjaak Philipsen, Guntram Suske

**Affiliations:** 1 Institute of Molecular Biology and Tumor Research, Philipps-University of Marburg, Marburg, Germany; 2 Department of Cell Biology, Erasmus MC, Rotterdam, The Netherlands; Medical Research Council Human Genetics Unit, United Kingdom

## Abstract

SUMO modification of transcription factors is linked to repression of transcription. The physiological significance of SUMO attachment to a particular transcriptional regulator, however, is largely unknown. We have employed the ubiquitously expressed murine transcription factor Sp3 to analyze the role of SUMOylation *in vivo*. We generated mice and mouse embryonic fibroblasts (MEFs) carrying a subtle point mutation in the SUMO attachment sequence of Sp3 (IKEE_553_D mutation). The E_553_D mutation impedes SUMOylation of Sp3 at K_551_ *in vivo*, without affecting Sp3 protein levels. Expression profiling revealed that spermatocyte-specific genes, such as *Dmc1* and *Dnahc8*, and neuronal genes, including *Paqr6*, *Rims3*, and *Robo3*, are de-repressed in non-testicular and extra-neuronal mouse tissues and in mouse embryonic fibroblasts expressing the SUMOylation-deficient Sp3E_553_D mutant protein. Chromatin immunoprecipitation experiments show that transcriptional de-repression of these genes is accompanied by the loss of repressive heterochromatic marks such as H3K9 and H4K20 tri-methylation and impaired recruitment of repressive chromatin-modifying enzymes. Finally, analysis of the DNA methylation state of the *Dmc1*, *Paqr6*, and *Rims3* promoters by bisulfite sequencing revealed that these genes are highly methylated in *Sp3wt* MEFs but are unmethylated in *Sp3E_553_D* MEFs linking SUMOylation of Sp3 to tissue-specific CpG methylation. Our results establish SUMO conjugation to Sp3 as a molecular beacon for the assembly of repression machineries to maintain tissue-specific transcriptional gene silencing.

## Author Summary

Cell type–specific gene expression patterns are largely regulated by positively or negatively acting transcription factors binding to promoter and enhancer elements. The ubiquitous transcription factor Sp3 represents a paradigm for a dual function transcription factor as it can activate and repress transcription. The repression function of Sp3 is mediated by attachment of a small protein designated SUMO to a single lysine residue. SUMOylation of Sp3 thus acts as a molecular switch that determines whether Sp3 acts as an activator or repressor. In this study, we have generated mice with a subtle mutation in the SUMO attachment site of Sp3. We found that several spermatocyte- and brain-specific genes that are silenced in non-testicular and extra-neuronal tissues of wild-type animals become aberrantly de-repressed in mice in which the SUMO attachment site of Sp3 is mutated. De-repression of these genes is accompanied with dramatic epigenetic changes including the loss of repressive histone methylation marks and, most significantly, loss of DNA methylation. Our findings suggest that SUMO modification of a transcription factor can act as a molecular beacon for the assembly of repression machineries to maintain tissue-specific transcriptional gene silencing *in vivo*.

## Introduction

A plethora of proteins involved in regulating gene expression such as promoter-specific transcription factors, cofactors and chromatin-modifying enzymes are reversibly modified by the Small Ubiquitin-like MOdifier SUMO (reviewed in [1], [2]). With few exceptions, SUMO modification of transcriptional regulators correlates with repression of transcription [3]–[5].

**Figure 1:**
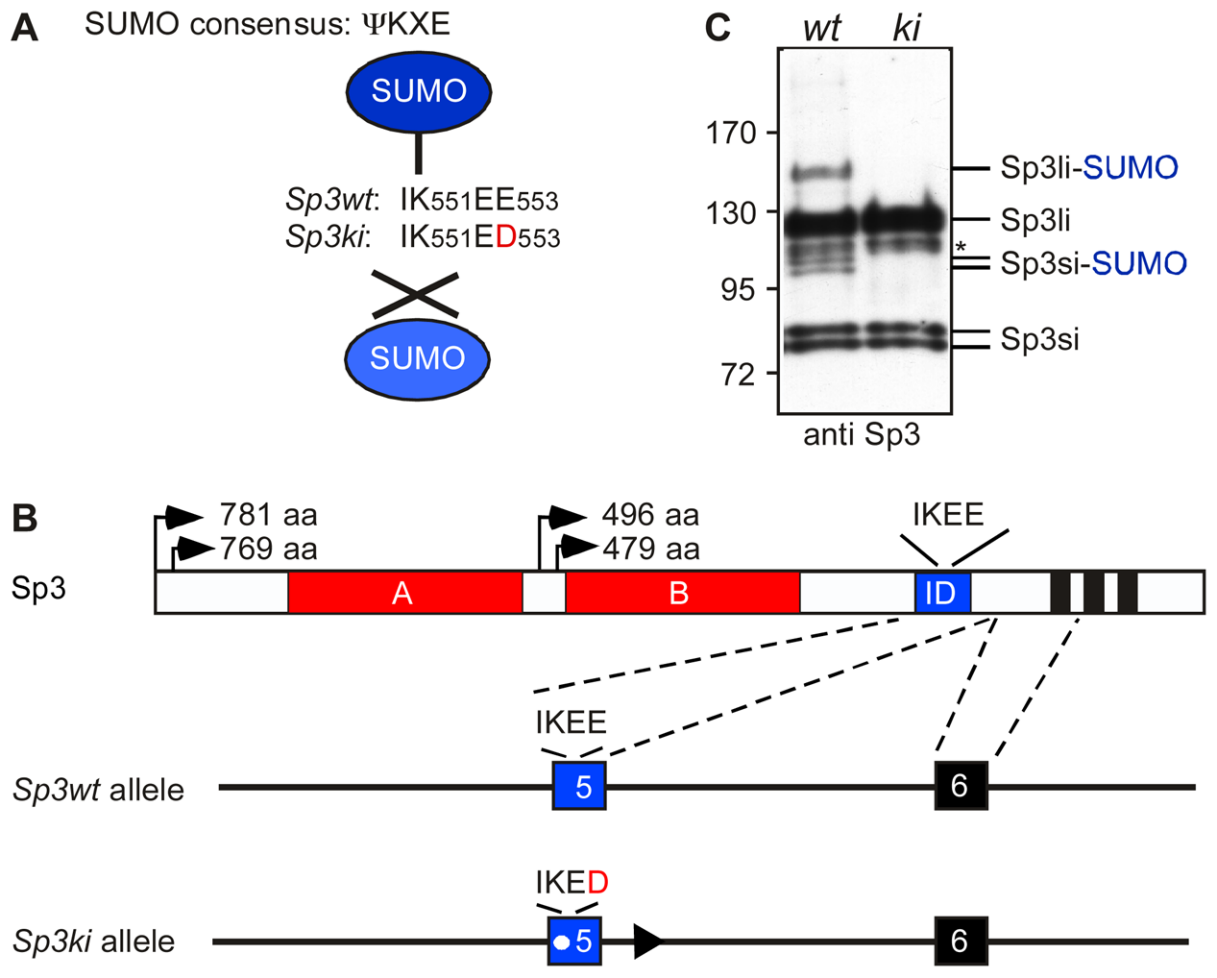
Targeting of the mouse Sp3 SUMO attachment site. (A) Sp3 is posttranslationally modified at K_551_ within the SUMO consensus sequence ΨKXE. In *Sp3ki/ki* mice the IK_551_EE_553_ sequence is replaced by IK_551_ED_553_. (B) Schematic presentation of Sp3 protein structure and part of the Sp3 gene. The glutamine-rich activation domains A and B, the SUMO attachment site (IKEE) within the inhibitory domain (ID) and the zinc fingers (black bars) of the DNA-binding domain are indicated. Arrows depict the four translational start sites and the lengths of the resulting Sp3 isoforms are shown. In the mutated *Sp3ki* allele, wild type exon 5 is replaced by the mutant exon 5 carrying the E_553_D mutation. (C) *Sp3ki/ki* mice lack SUMO modification of Sp3. MEFs obtained from *Sp3wt* (*wt*) and *Sp3ki/ki* (*ki*) embryos at E13.5 were subjected to Western blot analysis with Sp3 antibodies. The asterisk denotes an aspecific band.

The ubiquitously expressed transcription factor Sp3 represents a well-studied paradigm for regulation of activity by SUMOylation [6]–[8]. Sp3 belongs to the Sp (specificity protein) family of transcription factors that is implicated in the expression of a wide variety of genes including housekeeping, tissue-specific, developmentally and cell-cycle regulated genes [9]–[12]. A major feature of Sp3 is that, depending on promoter context, it can either activate or repress transcription in reporter gene assays [6], [7], [13]. Two glutamine-rich domains are known to exercise the activation function of Sp3 [13]; whereas the repressive activity of Sp3 is mediated by attachment of SUMO to lysine 551. K_551_ lies within the SUMO consensus motif IKEE located between the second Q-rich activation domain and the DNA-binding domain [6], [7] (Figure 1A and 1B). The functional complexity of Sp3 is further increased *in vivo* by the expression of four different isoforms that differ in their N-terminal extension [14]. All of these isoforms are SUMO-modified at K_551_, giving rise to a composite pattern of at least eight distinct protein species [14].

Previous investigations of the molecular events associated with Sp3-SUMO-dependent repression have provided mechanistic clues underlying SUMO-dependent gene silencing [15], [16]. SUMO-modification can act as a signal for the recruitment of various chromatin-associated repression components including the chromatin remodeler Mi-2, the MBT-domain proteins L3MBTL1 and L3MBTL2, heterochromatin protein 1 (HP1) and the histone methyltransferases (HMTs) SETDB1/ESET and SUV4-20H, concomitant with the establishment of repressive histone modifications such as H3K9 and H4K20 tri-methylation [16].

Despite extensive studies on the repression function of SUMOylated Sp3 and other transcription factors, the significance of SUMO attachment for the expression of endogenous genes *in vivo* is still largely unknown. Here, we report the generation of mice and mouse embryonic fibroblasts (MEFs) with a point mutation in the SUMO attachment sequence of Sp3. Expression profiling revealed that SUMOylation of Sp3 is required for silencing of spermatocyte-specific genes such as *Dmc1* and *Dnahc8* in somatic cells, and neuronal genes including *Paqr6*, *Rims3* and *Robo3* in non-neuronal cells. Transcriptional de-repression of these genes in MEFs expressing the Sp3E_553_D mutant protein is accompanied by the loss of repressive heterochromatic marks such as H3K9 and H4K20 tri-methylation, impaired recruitment of repressive chromatin-modifying enzymes and loss of DNA methylation. Our results establish that SUMO-modification of Sp3 acts as a platform for the assembly of repression machineries to maintain tissue-specific transcriptional gene silencing.

## Results

### Generating mutant mice deficient for SUMO modification of Sp3

To investigate the *in vivo* function of Sp3 SUMOylation, we generated mice in which the SUMO attachment site IK_551_EE is mutated to IK_551_ED (Figure 1A). We chose glutamic acid residue E_553_ for mutation because K_551_ might also be a target for other posttranslational modifications such as methylation or acetylation. A vector carrying the Sp3E_553_D mutation and a floxed neomycin-resistance cassette was used for targeting of ES cells to generate heterozygous mutant mice (Figure 1B; Figure S1). The neomycin-resistance gene was subsequently removed by mating with appropriate Cre recombinase-expressing mice [17]. Mice carrying the Sp3E_553_D mutation will from hereon be referred to as Sp3 knockin (*Sp3ki*) mutant. Heterozygous *Sp3wt/ki* mice and homozygous *Sp3ki/ki* mice were fertile, born at the expected Mendelian frequency and exhibited no obvious phenotype (Table S1).

To ensure that the E_553_D mutation impaired SUMOylation of Sp3, we performed Western blotting of adult mouse tissues and MEFs derived from E13.5 embryos. SUMO-modification of Sp3 was readily detectable in *Sp3wt* and heterozygous *Sp3wt/ki* but not in homozygous *Sp3ki/ki* tissues and MEFs (Figure 1C; Figure S1). This result demonstrates that the glutamic acid residue within the SUMOylation consensus motif ΨKXE is absolutely essential for the attachment of SUMO to endogenous Sp3. We conclude that the E_553_D mutation carried by *Sp3ki/ki* mice impedes SUMOylation of Sp3 at K_551_ *in vivo*, without affecting Sp3 protein levels.

### SUMOylated Sp3 represses spermatocyte-specific and neuronal genes

To identify genes that are regulated by SUMO-modified Sp3, we performed gene expression profiling with RNA extracted from primary *Sp3wt* and *Sp3ki/ki* MEFs derived from E13.5 littermates. This identified 68 genes that were upregulated and 7 genes that were downregulated by more than 2-fold in *Sp3ki/ki* MEFs (Table S2). Notably, top candidate genes that were upregulated in *Sp3ki/ki* MEFs encode developmentally-regulated meiotic and neuronal proteins. *Dmc1* and *Dnahc8* are expressed in meiotic spermatocytes and encode a RecA-like recombinase and a flagellar protein, respectively [18], [19]. *Paqr6*, *Rims3* and *Robo3* are expressed in the central nervous system [20]–[22]. The expression pattern of another upregulated gene (*Villin-like, Vill*) is largely unknown, although a low level of expression in early embryogenesis was reported [23].

**Figure 2:**
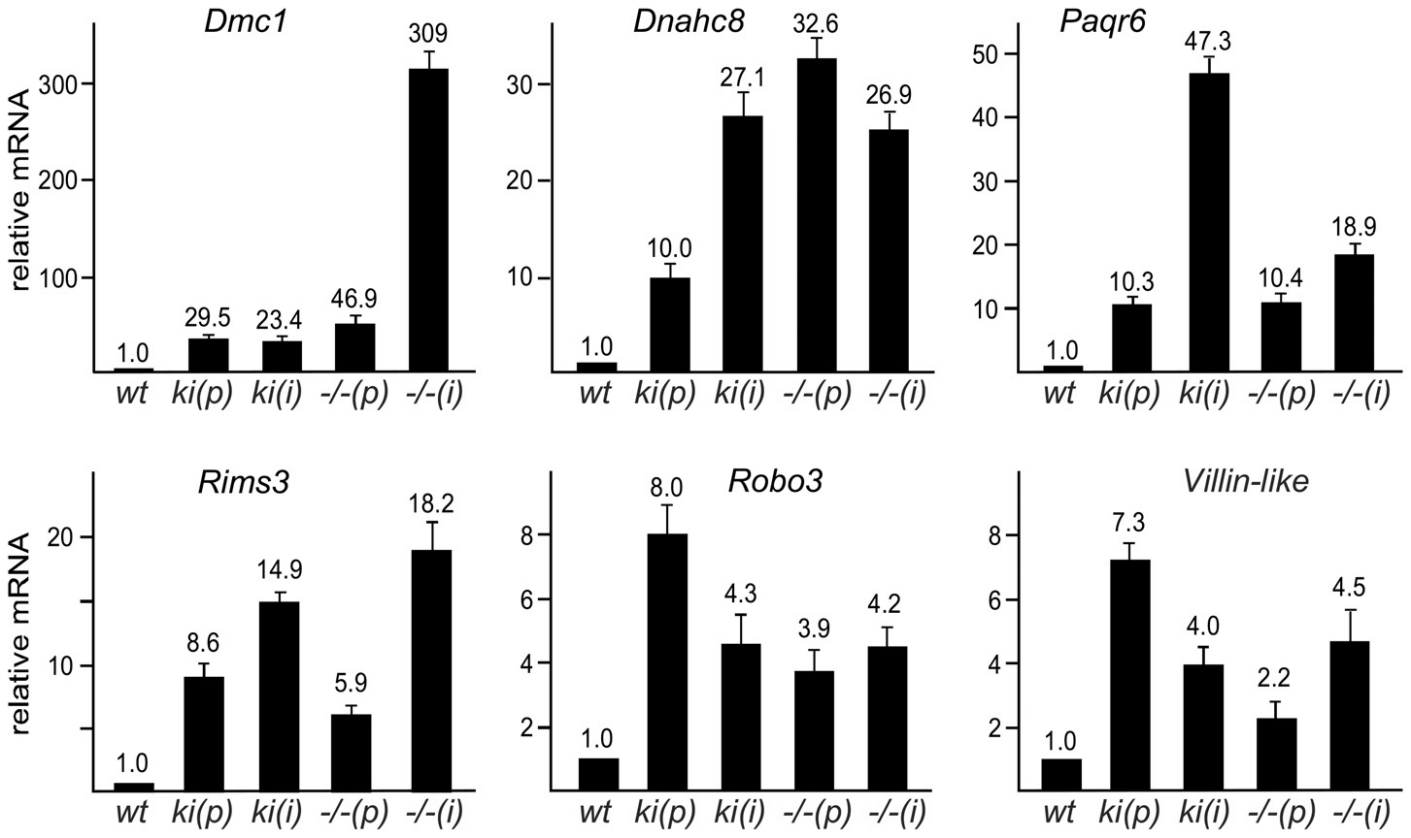
SUMOylation of Sp3 is required for silencing of spermatocyte-specific and neuronal genes in MEFs. Quantitative real-time RT-PCR analysis of *Dmc1*, *Dnahc8*, *Paqr6*, *Rims3*, *Robo3* and *Vill* expression in primary (*p*) and immortalized (*i*) *Sp3wt* (*wt*), *Sp3ki/ki* (*ki*) and Sp3-/- (*−/−*) MEFs. Normalized mRNA levels are presented relative to wild type MEFs arbitrarily set to 1. Data are expressed as mean +/−SD.

To validate aberrant de-repression of these genes, we analyzed their expression in MEF cultures by quantitative RT-PCR. *Dmc1*, *Dnahc8*, *Paqr6*, *Rims3*, *Robo3* and *Vill* mRNA levels were elevated in primary *Sp3ki/ki* MEFs as well as in immortalized *Sp3ki/ki* MEFs (Figure 2). We also analyzed expression of *Dmc1*, *Dnahc8*, *Paqr6*, *Rims3*, *Robo3* and *Vill* in Sp3-deficient (*Sp3-/-*) MEFs obtained from E13.5 Sp3 knockout embryos [24]. Consistent with an Sp3-SUMO-dependent silencing function, all six genes were upregulated also in *Sp3-/-* MEFs as compared to corresponding *Sp3wt* MEFs derived from littermates (Figure 2). We also performed immunoblot analysis for Dmc1 and Dnahc8 but failed to detect these proteins probably due to their low expression levels. However, de-repression of the *Dmc1* gene in *Sp3ki/ki* and *Sp3-/-* MEFs was verified with different amplimers spanning different exons (data not shown), thereby precluding the possibility that the qRT-PCR analyses detected an aberrant *Dmc1* transcript.

**Figure 3:**
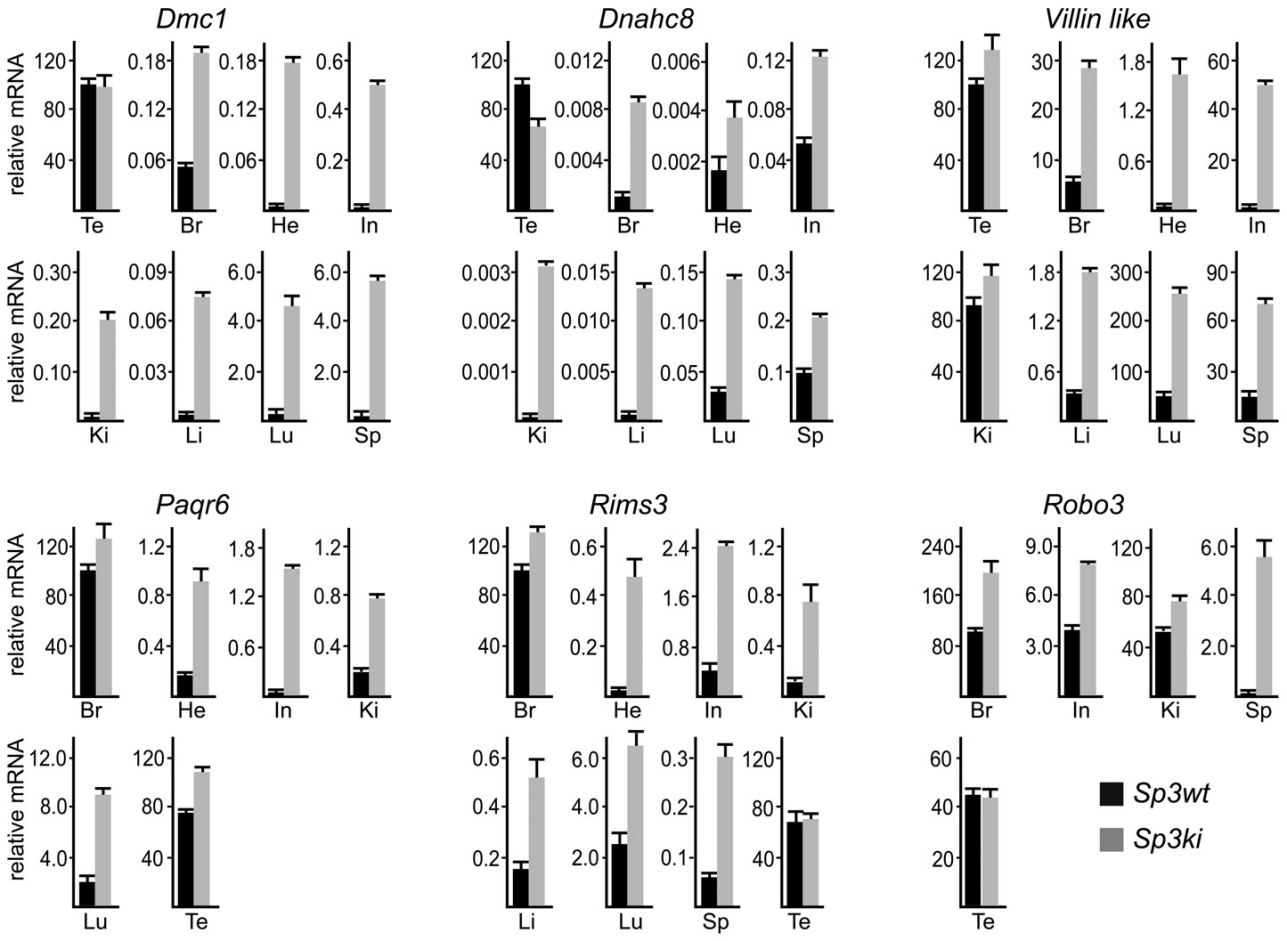
SUMOylation of Sp3 restricts tissue-specific gene expression in mouse organs. RNA from adult mouse organs was analyzed for *Dmc1, Dnahc8, Villin-like, Paqr6, Rims3* and *Robo3* expression by qRT-PCR. Expression levels were normalized to *Gapdh* mRNA and are presented relative to testis (*Dmc1, Dnahc8* and *Villin-like*) or brain (*Paqr6, Rims3* and *Robo3*) RNA arbitrarily set to 100. Abbreviations: Br, brain; Te, testis; He, heart; In, intestine; Ki, kidney; Li, liver, Lu, lung; Sp, spleen. Data are expressed as mean +/−SD.

De-repression of *Dmc1*, *Dnahc8*, *Paqr6*, *Rims3*, *Robo3* and *Vill* in *Sp3ki/ki* and *Sp3-/-* MEF cultures suggested that SUMO modification of Sp3 might be essential for silencing these genes in tissues other than testis and brain, respectively. We analyzed RNA from various tissues of adult *Sp3wt* and *Sp3ki/ki* mice. As expected, *Dmc1* and *Dnahc8* were strongly expressed in testis of *Sp3wt* and *Sp3ki/ki* mice at similar levels but were not or only marginally expressed in other tissues such as brain, heart, intestine, kidney, liver, lung and spleen. In *Sp3ki/ki* mice, *Dmc1* and *Dnahc8* mRNA levels were significantly higher in all tissues (Figure 3). However, the amount of RNA in testis was still one to three orders of magnitude higher indicating that testis-specific activators further enhance expression of *Dmc1* and *Dnahc8* in spermatocytes.

Previous reports have attributed silencing of several meiotic and male germ-line-specific genes in somatic cells such as *Smc1ß* and *Stag3* to E2F6 [25], [26], a repressive member of the E2F family of transcription factors. We analyzed expression of *Smc1ß* and *Stag3* in tissues of *Sp3ki/ki* mice as well. Both genes were only detectable in testis RNA preparations and were not de-repressed in non-testicular *Sp3ki/ki* tissues or *Sp3ki/ki* MEFs (data not shown). Vice versa, the *Dmc1* gene is not de-repressed in *E2F6-/-* MEFs [27] although the *Dmc1* promoter region is bound by E2F6 *in vivo* at a conserved binding site [27]. This observation suggests that different transcription factors and mechanisms are responsible for repressing spermatocyte-specific genes in somatic cells.

Consistent with published data, *Paqr6*, *Rims3* and *Robo3* mRNA levels were highest in mouse brain. Strikingly, all three genes were also highly expressed in testis and in the case of *Robo3* also in kidney. In all other organs these mRNAs were either not detectable or expressed only at a very low level (Figure 3). Nevertheless, expression of *Paqr6*, *Rims3* and *Robo3* as well as *Vill* mRNA was significantly elevated in several organs of *Sp3ki/ki* mice (Figure 3). Taken together, these results demonstrate that SUMOylation of Sp3 is essential for silencing of a subset of spermatocyte-specific and neuronal genes in somatic and non-neuronal tissues, respectively, implying an important role of the SUMO moiety attached to Sp3 in establishing tissue-specific gene expression patterns.

### Gene silencing of meiotic and neuronal genes is rescued by re-expression of SUMOylation-competent Sp3 in *Sp3-/-* MEFs

**Figure 4:**
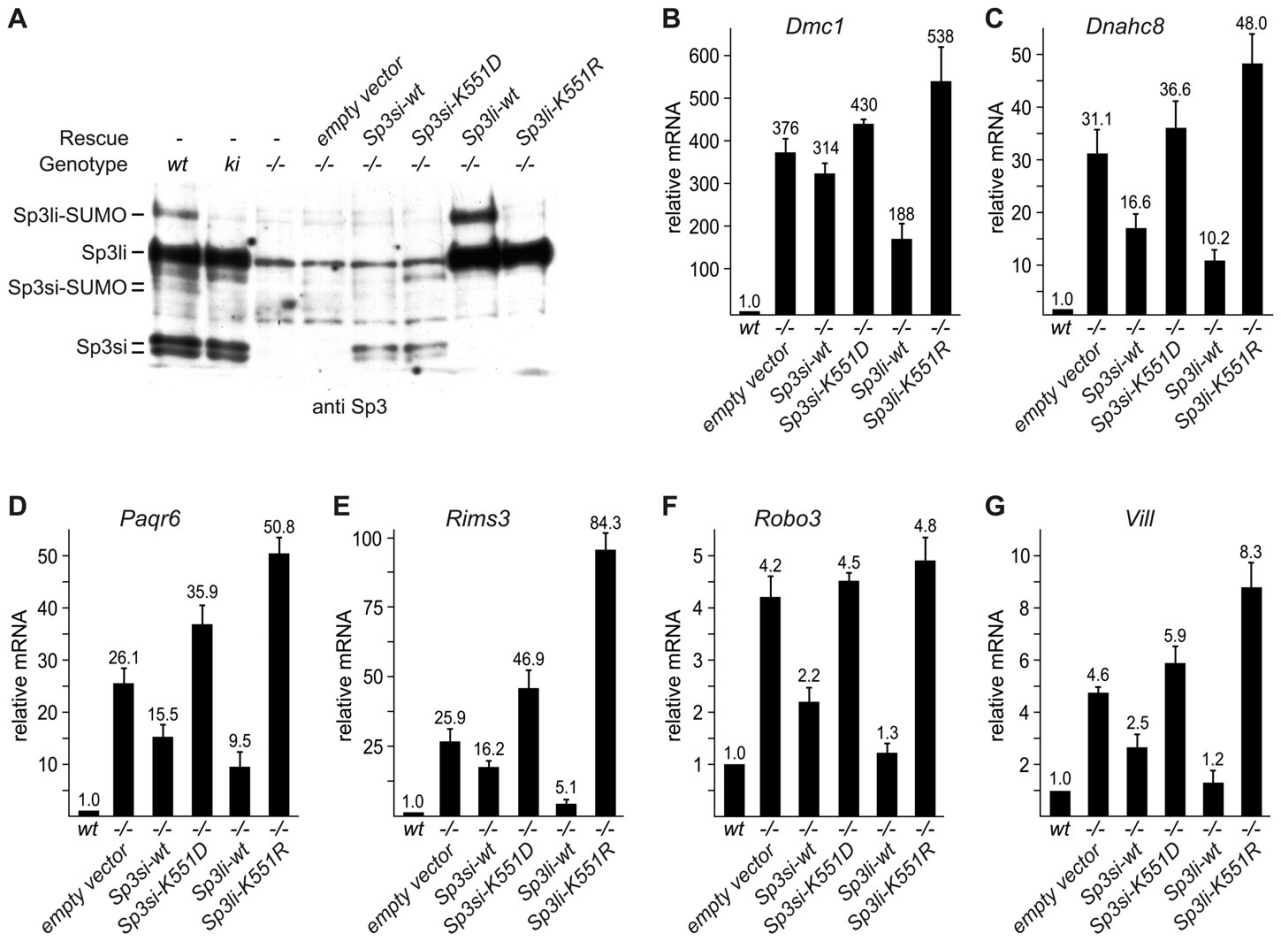
Sp3-SUMO–dependent rescue of gene silencing. *Sp3-/-* MEFs were transduced with retroviral expression vectors for the SUMOylation-competent long and short Sp3 isoforms (*Sp3li-wt* and *Sp3si-wt*), and corresponding SUMOylation-deficient mutants (*Sp3li-K_551_R* and *Sp3si-K_551_D*), respectively. (A) Immunoblot analysis of the various MEF cultures. (B–G) Expression of *Dmc1*, *Dnahc8, Paqr6, Robo3, Rims3 and Vill* in rescue MEFs. *Gapdh*-normalized mRNA expression levels are presented relative to the expression in wild type MEFs arbitrarily set to 1. Data are expressed as mean +/−SD.

To substantiate the notion that SUMOylation of Sp3 is directly responsible for silencing testis- and neuronal-specific genes in MEFs, we re-expressed the short and long isoforms of Sp3 (Sp3si-wt and Sp3li-wt) in *Sp3-/-* MEFs by retroviral transduction (Figure 4A). As controls, we used the corresponding SUMOylation-deficient Sp3 mutants (Sp3si-K_551_D and Sp3li-K_551_R). Particularly, re-expression of the long isoform of Sp3 resulted in significantly reduced expression of the *Dmc1*, *Dnahc8*, *Paqr6*, *Rims3*, *Robo3* and *Vill* genes. Repression of these genes by re-expression of the small isoforms of Sp3 was less pronounced (Figure 4B–4G). The weaker effects observed with the small isoforms of Sp3 could be due to their lower expression level (see Figure 4A). Potentially, simultaneous expression of all four wild type Sp3 isoforms would be necessary to restore repression completely. Nevertheless, in contrast to the wild type Sp3 isoforms, introduction of the SUMOylation-deficient Sp3 mutants failed to rescue gene silencing but instead further enhanced expression of these genes (Figure 4B–4G). These results show that reintroduction of Sp3 partially reverses de-repression of these genes in a SUMOylation-dependent manner.

### Sp3 is bound to the promoters of repressed genes *in vivo*

**Figure 5:**
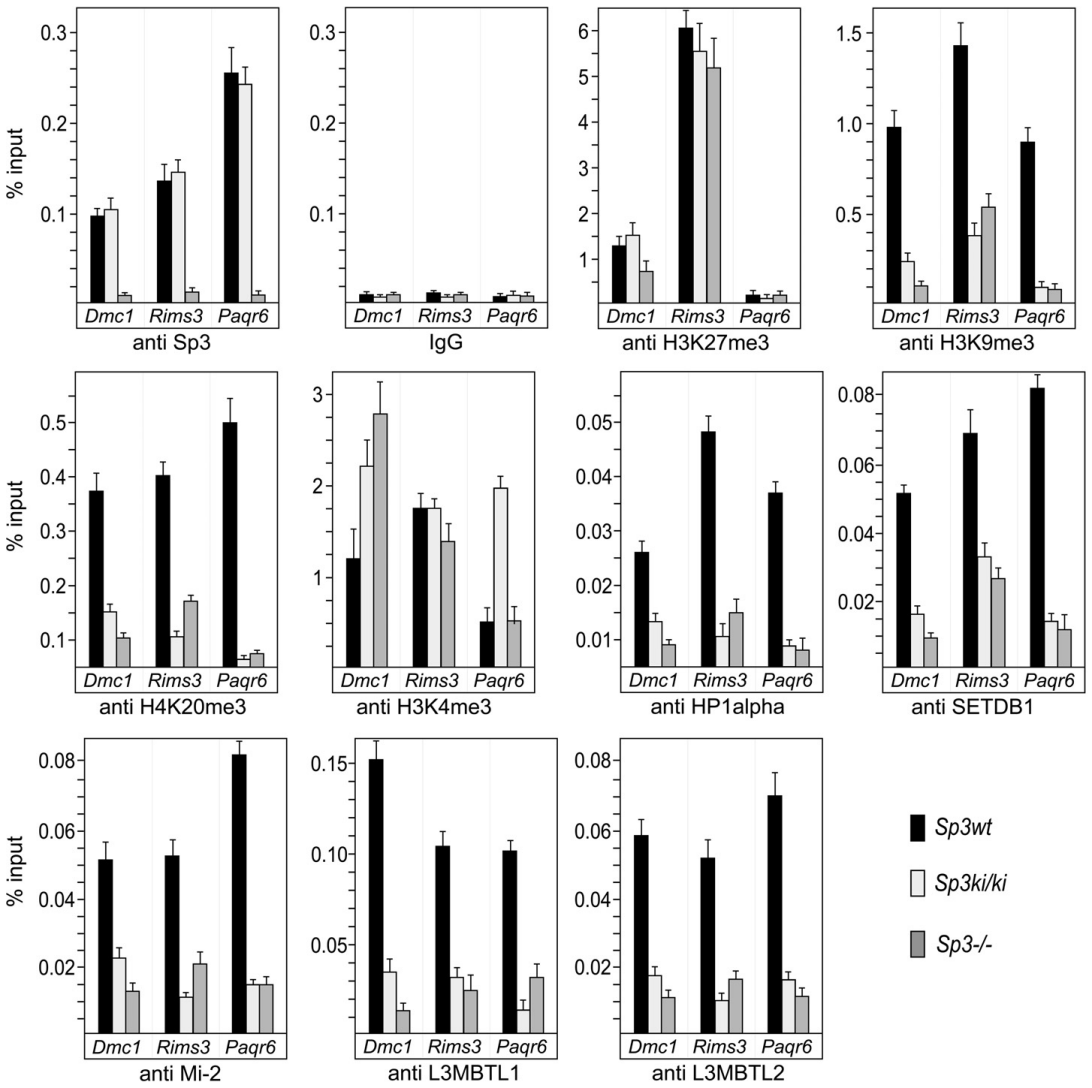
Sp3-SUMO–dependent association of heterochromatic histone modifications and repressive chromatin components. *Sp3wt*, *Sp3ki/ki* and *Sp3-/-* MEFs were subjected to ChIP analysis with the indicated antibodies. Precipitated DNA was amplified by qPCR with promoter-specific primers for *Dmc1*, *Rims3* and *Paqr6*. The locations of the primers are indicated as filled arrowheads in the schematic presentation of the promoters in Figure 6. DNA recoveries are expressed as percentage of input (mean +/−SD).

To analyze whether Sp3 is bound to the promoters of genes that are repressed by Sp3-SUMO, we performed ChIP analyses. Because of the lack of precise promoter information for *Dnahc8*, *Robo3* and *Vill* we focused on *Dmc1*, *Paqr6* and *Rims3*. All three promoters contain several potential binding sites for Sp3. Antibodies to Sp3 precipitated all three promoters from *Sp3wt* and *Sp3ki/ki* MEF chromatin but not from *Sp3-/-* MEF chromatin (Figure 5), demonstrating that both wild type Sp3 and the SUMOylation-deficient Sp3E_553_D mutant were bound to the *Dmc1*, *Paqr6* and *Rims3* promoters.

### Repressive chromatin components are enriched on promoters silenced by Sp3-SUMO

Next, we analyzed the *Dmc1*, *Paqr6* and *Rims3* promoters for the presence of repressive histone modifications (Figure 5). In *Sp3wt* MEFs, the H3K27me3 mark is abundantly present at the *Dmc1* and *Rims3* promoters but not at the *Paqr6* promoter. Moreover, this mark is not or only marginally reduced in the absence of SUMOylated Sp3 suggesting that H3K27me3 does not contribute to SUMO-dependent gene silencing of these three genes. In contrast, H3K9me3 and H4K20me3 marks are present at all three promoters in *Sp3wt* MEFs but are strongly reduced in *Sp3ki/ki* and in *Sp3-/-* MEFs. Consistently, HP1α, which binds H3K9me3, is present at the *Dmc1*, *Paqr6* and *Rims3* promoters in *Sp3wt* MEFs but not in *Sp3ki/ki* and *Sp3-/-* MEFs. Thus, the presence or absence of H3K9me3, H4K20me3 and HP1α at the *Dmc1*, *Paqr6* and *Rims3* promoters correlates strictly with the repressed or de-repressed state of these genes. We also analyzed for the presence of H3K4 trimethylation, an epigenetic mark characteristic for promoter-proximal nucleosomes of most active as well as inactive genes [28]. The co-occurrence of H3K9me3, H3K27me3 and H3K4me3 marks is a characteristic property of “bivalent” promoters of euchromatic genes in ES cells, believed to reflect a repressed but poised transcriptional state [29]. The H3K4me3 mark was abundantly present on the *Dmc1*, *Paqr6* and *Rims3* promoters in *Sp3wt* MEFs. In *Sp3ki/ki* MEFs we found an approximately 2-fold and 3-fold increase of H3K4 trimethylation on the *Dmc1* promoter and on the *Paqr6* promoter, respectively, but not on the *Rims3* promoter (Figure 5). In *Sp3-/-* cells, higher H3K4me3 levels were detected on the *Dmc1* promoter but not on the *Rims3* and *Paqr6* promoters. Thus, there is no strict correlation between the changes of the H3K4me3 mark and the expression state of the different target genes.

Our previous investigations revealed that the establishment of repressive nucleosomal signatures on a chromatinized Gal4-driven reporter gene by Gal4-Sp3-SUMO involves the recruitment of the histone methyltransferase SETDB1, the chromatin remodeler Mi-2, and the chromatin-compacting MBT-domain proteins L3MBTL1 and L3MBTL2 [16]. Therefore, we analyzed the *Dmc1*, *Paqr6* and *Rims3* promoters for the presence of these proteins. ChIP analysis revealed that all four proteins are abundantly present on the promoters in *Sp3wt* MEFs but strongly reduced in *Sp3ki/ki* and *Sp3-/-* MEFs (Figure 5). The recruitment of these chromatin-modifying proteins to the endogenous *Dmc1*, *Paqr6* and *Rims3* promoters is thus dependent on the presence of the Sp3 SUMO moiety. Taken together, these results support the conclusion that the posttranslational modification of Sp3 at K_551_ provokes a local repressive chromatin structure on a subset of spermatocyte- and neuronal-specific genes in somatic and non-neuronal cells, respectively.

Repression of many nervous system-specific genes in unrelated tissues has been attributed to the corepressor CoREST1. CoREST1 is recruited to promoters by the transcriptional repressor REST/NRSF [30] and, alternatively, by a REST/NRSF-independent mechanism that involves direct interaction between CoREST1 and a thus far unknown SUMO2/3-modified transcription factor [31]. To examine whether CoREST1 is also involved in silencing the *Dmc1*, *Paqr6* and *Rims3* promoters, we performed ChIP analysis for the presence of CoREST1. CoREST1 was to some extent detectable on the *Rims3* promoter but not on the *Dmc1* and *Paqr6* promoters irrespectively of the Sp3 SUMOylation status (Figure S2). This finding indicates that SUMOylation of Sp3 represents an alternative, CoREST1-independent pathway mediating extra-neuronal repression.

### SUMO modification of Sp3 is essential for DNA methylation of target genes

**Figure 6:**
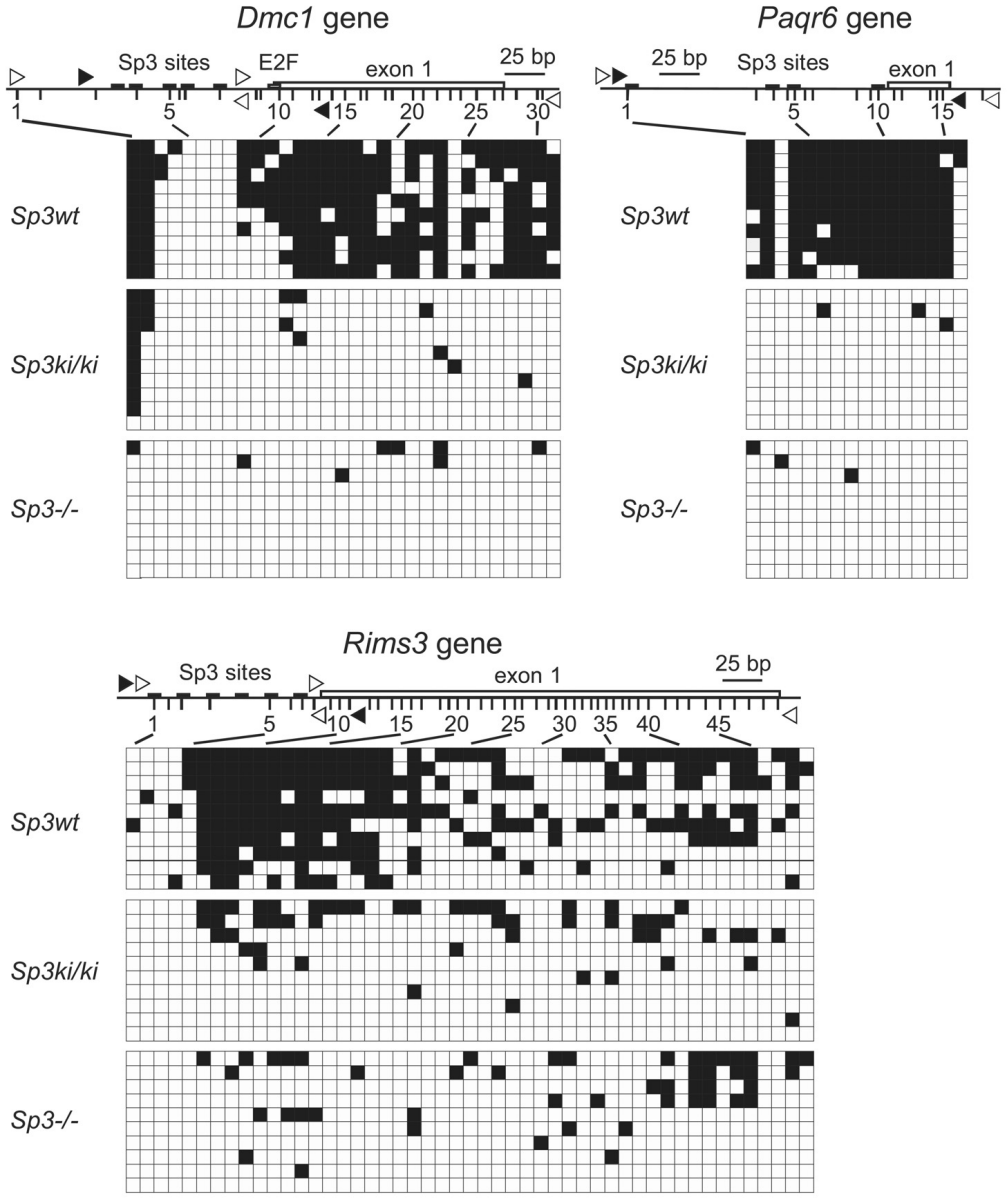
SUMOylation-dependent CpG methylation of the *Dmc1*, *Paqr6*, and *Rims3* genes. The methylation state of individual CpGs in *Sp3wt*, *Sp3ki/ki* and *Sp3-/-* MEFs was analyzed by bisulfite sequencing of ten clones. Non-methylated CpGs are indicated as open squares and methylated CpGs as filled squares. The promoters along with the position of Sp3 binding sites and the first exon are depicted schematically. Vertical lines depict the positions of CpG dinucleotides. Unfilled arrowheads indicate the locations of the primers used for PCR, and filled arrowheads indicate the locations of the primers used for the ChIP experiments shown in Figure 5.

An EMBOS CpGPlot analysis (http://www.ebi.ac.uk/Tools/emboss/cpgplot/index.html) revealed that the *Dmc1*, *Paqr6* and *Rims3* promoters are embedded in CpG islands. CpG island methylation may contribute to silencing of these genes in MEFs and may be reversed in *Sp3ki/ki* and *Sp3-/-* MEFs. Therefore, we analyzed the methylation states of these promoters in *Sp3wt*, *Sp3ki/ki* and *Sp3-/-* MEFs. Bisulfite sequencing revealed that the proximal promoters and the first exons of the *Dmc1*, *Paqr6* and *Rims3* genes are highly methylated in *Sp3wt* MEFs. In contrast, CpG methylation is strongly reduced in *Sp3ki/ki* and in *Sp3-/-* MEFs (Figure 6). In summary, there is a very tight correlation between SUMO modification of Sp3, transcriptional repression, repressive histone modifications and DNA methylation of these promoters. Lack of SUMOylation of Sp3 results in their de-repression accompanied by the absence of repressive histone modifications and the absence of CpG methylation.

## Discussion

The generation and analysis of mice with a subtle point mutation in the SUMO attachment site of the transcription factor Sp3 has revealed the relevance of SUMO modification of Sp3 for gene silencing *in vivo*. SUMO attachment to Sp3 serves as a molecular beacon for the recruitment of chromatin-modifying machineries that impose epigenetic silencing on a subset of spermatocyte-specific and neuronal genes. Our data are consistent with the bidirectional crosstalk between repressive histone modification and DNA methylation, that was established by demonstrating direct interactions between SETDB1 and the *de novo* methylase DNMT3A [32]. However, SETDB1 may also be recruited indirectly through interaction with the methyl-CpG binding protein MBD1 that forms a stable complex with SETDB1 [33]. We note that a functional SUMO interaction motif (SIM) is present in the histone methyltransferase SETDB1 [34] and that potential SIMs are also present in DNMTs. Thus, SUMO-modified Sp3 might recruit SETDB1 and DNMTs independently. Sp3-SUMO-mediated repression might involve a timely coordinated recruitment of chromatin remodelers, nucleosome compactors such as MBT domain proteins, HMTs and DNMTs. Alternatively, these different types of epigenetic players might be recruited simultaneously. The *Sp3ki/ki* and *Sp3-/-* MEFs described here provide essential tools for future experiments addressing these questions.

The lack of Sp3 SUMOylation in *Sp3ki/ki* mice causes aberrant expression of several spermatocyte-specific and neuronal genes in various somatic tissues. Although de-repression of these genes is best described by the loss of the repressive function of SUMOylated Sp3 in *Sp3ki/ki* mice, it is conceivable that the activation function of the Sp3E_553_D mutant protein may contribute directly or indirectly to the aberrant expression of these genes as well. However, expression of the spermatocyte-specific and neuronal genes in testis and brain, respectively, is not affected. This tissue-selectivity could be due to low-level Sp3 SUMOylation in spermatocytes and neurons. To investigate this, we performed Western blot analysis of testis and brain extracts. Consistent with the lack of repression in testis and brain, SUMOylated Sp3 species were barely detectable in these tissues (Figure S3). Future studies using purified spermatocytes and neurons might provide further insight on the cell type-specific SUMOylation state of Sp3. Interestingly, it has been reported that Sp3 expression in germ cells declines during the leptotene to pachytene transition whereas the related transcription factor Sp1 did not decline until the mid-pachytene phase of meiosis [35]. Dmc1 is expressed in leptotene to zygotene spermatocytes [19], and Dnahc8 from mid-pachytene to diplotene spermatocytes [18]. Thus, activation of the *Dmc1* and *Dnahc8* genes at these meiotic stages correlates with down-regulation of Sp3. Accordingly, one could image a scenario in which down-regulation of the Sp3 protein level facilitates Sp1-mediated activation of these two Sp3-SUMO target genes during spermatocyte development.

The promoters of the three Sp3-SUMO target genes that we analyzed in detail share several features. They contain multiple GC-boxes, lack a TATA box and are embedded in CpG islands. Such a promoter arrangement is reminiscent of housekeeping genes. In contrast to the three Sp3 target genes, housekeeping genes are ubiquitously expressed and remain unmethylated in all tissues. It is currently unclear why the three Sp3 target genes are expressed in such a highly tissue-specific manner. We have not detected obvious features in the spacing or orientation of Sp-binding sites that can account for this interesting difference. Further investigations comparing these two types of promoters are required to address this enigmatic point.

The aberrant expression of several testicular and neuronal genes in *Sp3ki/ki* mice apparently does not lead to obvious histological or behavioral abnormalities under standard mouse housing conditions indicating that essential functions of differentiated cell types are not grossly impaired. We note that the overall amounts of aberrantly produced mRNA transcripts in somatic extra-neuronal cells are low. This provides an explanation for the absence of clear anatomical and physiological anomalies in the *Sp3ki/ki* mice.

The phenotype of the *Sp3ki/ki* mice differs significantly from mice lacking the entire Sp3 protein. Sp3-deficient mice display skeletal, tooth, hematopoietic and heart defects at late embryonic development, and die immediately after birth due to respiratory failure [24], [36]–[38]. Given that SUMOylation-deficient Sp3 proteins are strong activators [13], [14] the defects observed in *Sp3-/-* mice have to be attributed largely to the activation function of Sp3.

## Materials and Methods

### Ethics statement

Research involving mice have been conducted according to the German Animal Protection Law (Tierschutzgesetz). The application for the experiments was reviewed and approved by the responsible local authorities (Regierungspräsidium Giessen, reference number V 54–19 c 20/15 cMR20/27).

### Generation of *Sp3ki/ki* mice

A targeting vector containing the Sp3E_553_D mutation was constructed and transfected into ES cells (Text S1). For selection of ES cells, we used a floxed *IRES-LacZ-neo-polyA* cassette that integrates into intron 5 of the *Sp3* gene by homologous recombination. A single clone out of >200 G418-resistant colonies showed the homologous recombination event. After karyotyping, the ES clone was injected into C57BL/6 blastocysts. Breeding of the chimeras revealed germ-line transmission of the targeted *Sp3* allele. The *IRES-lacZ-neo* cassette was removed by mating heterozygous mice with mice expressing the Cre recombinase under control of the cytomegalovirus-immediate early enhancer-chicken beta-actin hybrid (*CAG*) promoter [17]. Offspring were genotyped by Southern blotting and PCR (Figure S1). The *Sp3wt/ki* heterozygous offspring were intercrossed and homozygous *Sp3ki/ki* mice were obtained.

### Retroviral infections

Retroviral vectors for expression of the long and short isoforms of Sp3 and corresponding SUMOylation-deficient mutants were generated by cloning of appropriate wild type and mutant *Sp3* cDNA fragments [14] into the pBABE-puro plasmid. Retroviral packaging in Phoenix cells and infection of immortalized *Sp3-/-* MEFs [24] were performed according to standard procedures. Transduced cells were selected for uptake of retrovirus with 2 µg/mL of puromycin.

### Western blotting

Whole cell extract from MEFs and mouse tissues were prepared as described [7], separated on SDS-polyacrylamide gels, blotted on PVDF membranes and probed with anti Sp3 antibodies (Santa Cruz Biotechnology, sc-644). Secondary antibodies were visualized using the Immobilon Western HRP substrate (Millipore).

### Expression profiling

Total RNA was prepared from freshly isolated *Sp3wt* and *Sp3ki/ki* mouse embryonic fibroblasts of E13.5 siblings using the RNeasy kit (Qiagen). Purified RNA was labeled with the two-color Quick-Amp Labeling kit (Agilent) and hybridized to a whole genome microarray 4x44K 60mer slide (G4122F) according to the manufacturer's instructions (Agilent). Microarray data were analyzed using Bioconductor [39]. The loess method implemented in the Bioconductor package marray was applied for normalization. Two biological replicates (male and female MEFs) were performed. Genes were considered as regulated when they had a fold change of ≥2, a logarithmic intensity value (base 2) of ≥5 in *Sp3ki/ki*, and when the expression level of replicates were similar. Similarity for two log2 transformed expression levels was determined ad hoc by the constraint *max(1, |e1, e2| ×0.75,) > |e1 – e2|.*

### Data deposition

Microarray data were deposited at ArrayExpress (www.ebi.ac.uk/arrayexpress) under accession number E-MEXP-2755.

### Quantitative real-time PCR

One microgram of total RNA prepared from MEFs and mouse organs was used for cDNA synthesis along with 0.5 µg of oligo(dT) primer and 200 U of M-MLV reverse transcriptase (Invitrogen)**.** Quantitative RT-PCR was performed with 1 µL of 1∶20 diluted cDNA using gene-specific primers (Text S1). qPCRs were performed in quadruplicate using the Absolute SYBRGreen qPCR Mix (Abgene) on the Mx3000P real-time PCR system (Stratagene). Values were normalized to Gapdh and/or Sp1 mRNA content.

### Chromatin immunoprecipitation

Chromatin immunoprecipitation was performed using the One Day ChIP kit (Diagenode) in accordance to the manufacturer's instructions. Primer sequences specific for *Dmc1*, *Paqr6* and *Rims3* promoter regions can be found in Text S1. Antibodies used for ChIP analysis are described in [16].

### Bisulfite sequencing

For DNA methylation analysis, 2 µg of genomic DNA derived from immortalized *Sp3wt, Sp3-/-* and *Sp3ki/ki* MEFs were subjected to sodium bisulfite conversion of unmethylated cytosines using the EpiTect Bisulfite Kit (Qiagen) in accordance to the manufacturer's instructions. Converted DNA was subjected to PCR amplification using promoter-specific *BamHI-* and *KpnI-*tailed primers (Text S1) and the ImmoMix PCR reagent (Bioline). PCR products were cloned into the pcDNA3 vector and 10 clones were sequenced using the BGHrev primer.

## Supporting Information

Figure S1 Targeting the mouse Sp3 SUMO site. (A) Top, schematic representation of Sp3 protein structure. The glutamine-rich activation domains A and B, the SUMO attachment site (IKEE) within the inhibitory domain (ID) and the zinc fingers (black bars) of the DNA binding domain as well as the translational start sites and the length of the four different isoforms (781, 769, 496 and 479 aa) of Sp3 are indicated. Connecting lines with the corresponding murine *Sp3* gene regions show the derivation of the inhibitory domain of the Sp3 protein from exon 5. In the targeting vector, exon 5 was replaced by the mutagenized exon and a floxed cassette containing a splice acceptor site (*En2-SA*), an internal ribosomal entry site (*IRES*) and a *lacZ-neomycin* fusion gene (*lacZ-Neo*) [ Mountford, *et al*]. The positions of the BglII (B) sites, the probe used for Southern blotting (B-probe) and the lengths of the corresponding DNA fragments are given as well. (B) Restriction of genomic DNA with BglII and hybridization with the probe indicated in (A) detected a 6.7 kb fragment of the wild type allele and a 10.6 kb fragment of the mutated allele. (C) Schematic presentation of the *Sp3ki* allele after removal of the selection cassette by crossing targeted heterozygous mice with *CAG*-driven Cre recombinase expressing mice. (D) Southern blot analysis after EcoRV restriction revealed successful deletion of the selection cassette. (E) PCR strategy for genotyping of *Sp3ki* mice. The *Sp3wt* allele, the *Sp3ki* allele, the position of allele-specific primers (arrows) and the lengths of the amplicons are depicted. The black arrowhead in the *Sp3ki* allele represents the remaining *loxP* site after removal of the *lacZ-neo* cassette. (F) PCR analysis. Sequence-specific primers for the *Sp3wt* and the *Sp3ki* alleles and a common intronic reverse primer produce 728 bp and 762 bp DNA fragments, respectively. (G) Lack of Sp3 SUMOylation in tissues and MEFs of *Sp3ki/ki* mutant mice. Mouse lung and spleen tissue samples, and MEFs obtained from E13.5 embryos as well as from *Sp3wt* (*wt/wt*) and *Sp3ki/ki* mice (*ki/ki*) were subjected to immunoblot analyses with antibodies specific for Sp3. The asterisk indicates an aspecific band. [Mountford P, Zevnik B, Duwel A, Nichols J, Li M, et al. (1994) Dicistronic targeting constructs: reporters and modifiers of mammalian gene expression. Proc Natl Acad Sci USA 91: 4303-4307.]
(2.18 MB TIF)

Figure S2 Sp3-SUMO-dependent silencing of the *Dmc1*, *Paqr6* and *Rims3* promoters is independent of CoREST. *Sp3wt* (*wt*), *Sp3ki/ki* and *Sp3-/-* MEFs were subjected to ChIP analysis with a CoREST-specific antibody. The glutamate receptor *M4* promoter was used as positive control [30]. Precipitated DNA was amplified by qPCR with primers for the *M4*, *Dmc1, Paqr6* and *Rims3* promoters. DNA recoveries are expressed as percentage of input (mean +/− SD).
(0.09 MB TIF)

Figure S3 Sp3 expression in mouse testis and brain. Mouse testis, brain and lung protein samples from adult *Sp3wt* (*wt*) and *Sp3ki/ki* mice (*ki*) were subjected to immunoblot analyses with anti Sp3 antibodies. Two different exposure times of the blots are shown. The asterisks indicate uncharacterized Sp3 isoforms or aspecific bands. The SUMOylated small isoforms could not assigned to specific signals.
(1.02 MB TIF)

Table S1 Genotype distribution of *Sp3wt/ki* intercrossings.
(0.03 MB DOC)

Table S2 List of differentially expressed genes identified by expression profiling.
(0.03 MB XLS)

Text S1 Supporting materials and methods. Generation of the *Sp3* knockin homologous recombination construct. Genotyping of targeted Sp3 knockin mice by PCR. Transfection of ES cells and generation of chimeric and Sp3 SUMOylation-deficient mice. Primers for RT-qPCR. Primers for promoter/exon amplification and cloning after bisulfite treatment.
(0.04 MB DOC)
