# Supplementary figures and images for: Epigenetic Silencing of Spermatocyte-Specific and Neuronal Genes by SUMO Modification of the Transcription Factor Sp3
Source: PLoS Genet. 2010 Nov 11;6(11):e1001203. doi: 10.1371/journal.pgen.1001203 (PMC2978682; doi:10.1371/journal.pgen.1001203)

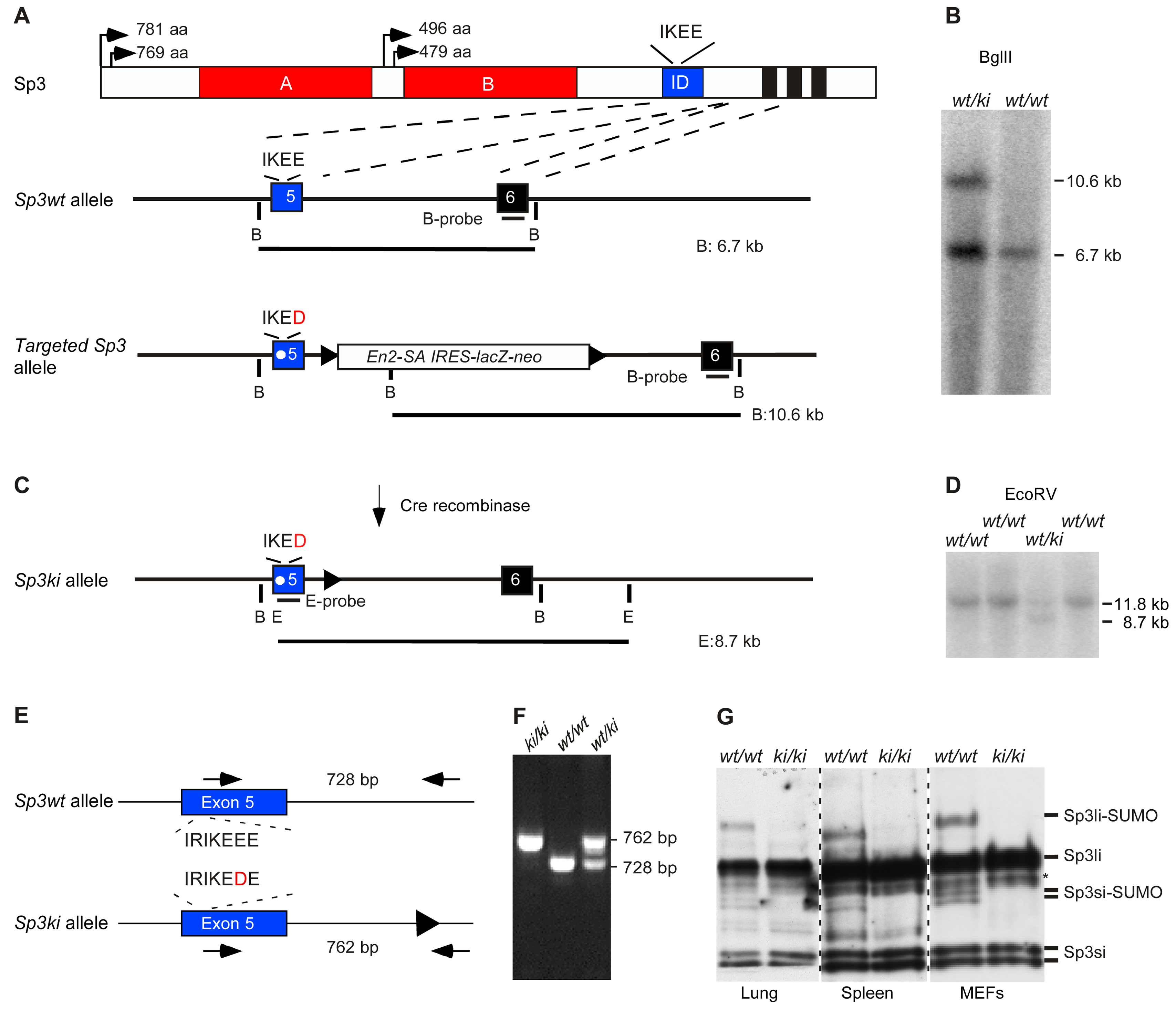

Supplement: Figure S1 — Targeting the mouse Sp3 SUMO site. (A) Top, schematic representation of Sp3 protein structure. The glutamine-rich activation domains A and B, the SUMO attachment site (IKEE) within the inhibitory domain (ID) and the zinc fingers (black bars) of the DNA binding domain as well as the translational start sites and the length of the four different isoforms (781, 769, 496 and 479 aa) of Sp3 are indicated. Connecting lines with the corresponding murine Sp3 gene regions show the derivation of the inhibitory domain of the Sp3 protein from exon 5. In the targeting vector, exon 5 was replaced by the mutagenized exon and a floxed cassette containing a splice acceptor site (En2-SA), an internal ribosomal entry site (IRES) and a lacZ-neomycin fusion gene (lacZ-Neo) [ Mountford, et al]. The positions of the BglII (B) sites, the probe used for Southern blotting (B-probe) and the lengths of the corresponding DNA fragments are given as well. (B) Restriction of genomic DNA with BglII and hybridization with the probe indicated in (A) detected a 6.7 kb fragment of the wild type allele and a 10.6 kb fragment of the mutated allele. (C) Schematic presentation of the Sp3ki allele after removal of the selection cassette by crossing targeted heterozygous mice with CAG-driven Cre recombinase expressing mice. (D) Southern blot analysis after EcoRV restriction revealed successful deletion of the selection cassette. (E) PCR strategy for genotyping of Sp3ki mice. The Sp3wt allele, the Sp3ki allele, the position of allele-specific primers (arrows) and the lengths of the amplicons are depicted. The black arrowhead in the Sp3ki allele represents the remaining loxP site after removal of the lacZ-neo cassette. (F) PCR analysis. Sequence-specific primers for the Sp3wt and the Sp3ki alleles and a common intronic reverse primer produce 728 bp and 762 bp DNA fragments, respectively. (G) Lack of Sp3 SUMOylation in tissues and MEFs of Sp3ki/ki mutant mice. Mouse lung and spleen tissue samples, and MEFs obta [file pgen.1001203.s001.tif]

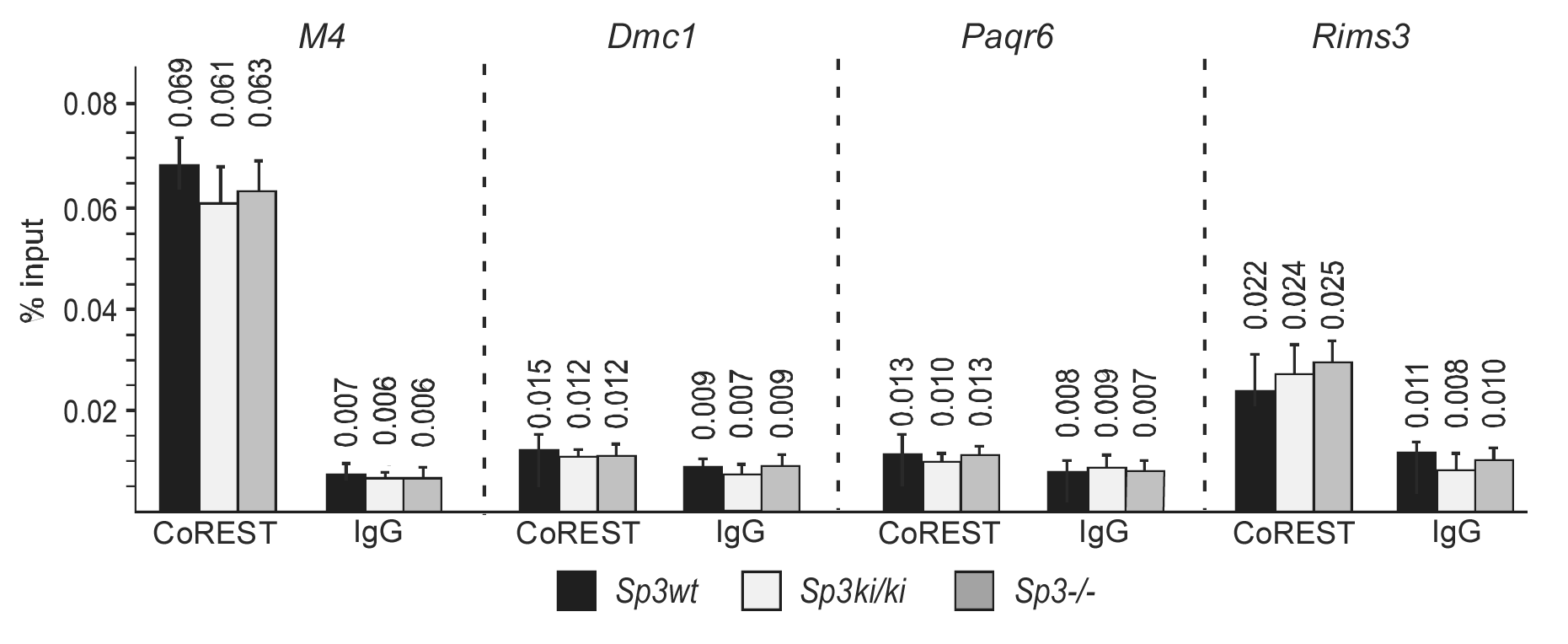

Supplement: Figure S2 — Sp3-SUMO-dependent silencing of the Dmc1, Paqr6 and Rims3 promoters is independent of CoREST. Sp3wt (wt), Sp3ki/ki and Sp3-/- MEFs were subjected to ChIP analysis with a CoREST-specific antibody. The glutamate receptor M4 promoter was used as positive control [30]. Precipitated DNA was amplified by qPCR with primers for the M4, Dmc1, Paqr6 and Rims3 promoters. DNA recoveries are expressed as percentage of input (mean +/− SD). (0.09 MB TIF) [file pgen.1001203.s002.tif]

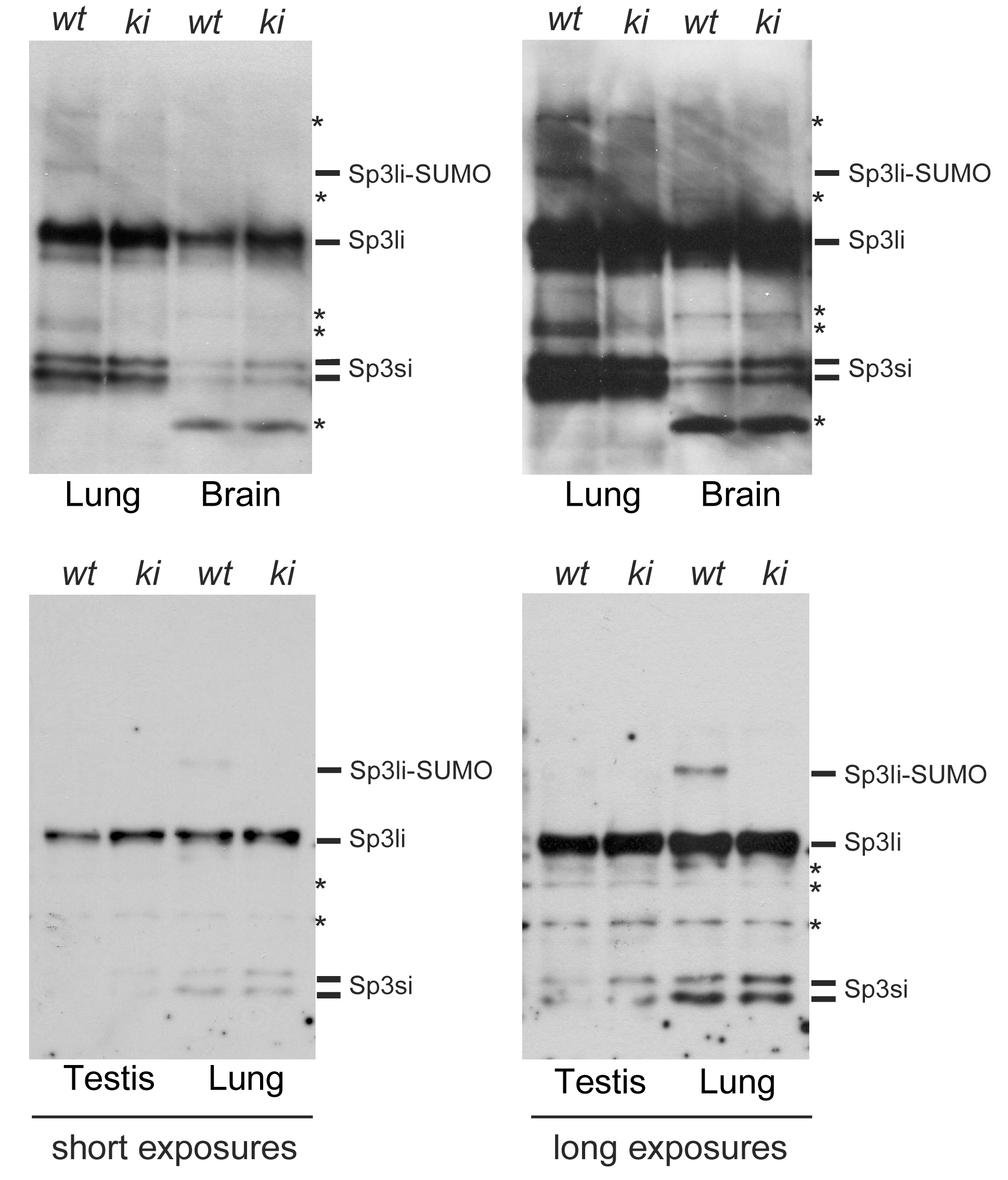

Supplement: Figure S3 — Sp3 expression in mouse testis and brain. Mouse testis, brain and lung protein samples from adult Sp3wt (wt) and Sp3ki/ki mice (ki) were subjected to immunoblot analyses with anti Sp3 antibodies. Two different exposure times of the blots are shown. The asterisks indicate uncharacterized Sp3 isoforms or aspecific bands. The SUMOylated small isoforms could not assigned to specific signals. (1.02 MB TIF) [file pgen.1001203.s003.tif]
