# Supplementary material for: Epigenetic Silencing of Spermatocyte-Specific and Neuronal Genes by SUMO Modification of the Transcription Factor Sp3
Source: PLoS Genet. 2010 Nov 11;6(11):e1001203. doi: 10.1371/journal.pgen.1001203 (PMC2978682; doi:10.1371/journal.pgen.1001203)
Supplement: Table S1 — Genotype distribution of Sp3wt/ki intercrossings. (0.03 MB DOC) [file pgen.1001203.s004.doc]

**Supporting Table**

**Stielow et al.**

**Table S1. Genotype distribution of *Sp3wt/ki* intercrossings**

| Sp3wt/ki x Sp3wt/ki | | | ***Sp3wt/wt*** | ***Sp3wt/ki*** | ***Sp3ki/ki*** |
| --- | --- | --- | --- | --- | --- |
| All  Male  Female | n=203  n=114  n= 89 | (100%)  (100%)  (100%) | 49 (24.1%)  24 (21.1%)  25 (28.1%) | 113 (55.7%)  68 (59.6%)  45 (50.6%) | 41 (20.2%)  22 (19.3%)  19 (21.3%) |
