# Supplementary material for: Epigenetic Silencing of Spermatocyte-Specific and Neuronal Genes by SUMO Modification of the Transcription Factor Sp3
Source: PLoS Genet. 2010 Nov 11;6(11):e1001203. doi: 10.1371/journal.pgen.1001203 (PMC2978682; doi:10.1371/journal.pgen.1001203)
Supplement: Text S1 — Supporting materials and methods. Generation of the Sp3 knockin homologous recombination construct. Genotyping of targeted Sp3 knockin mice by PCR. Transfection of ES cells and generation of chimeric and Sp3 SUMOylation-deficient mice. Primers for RT-qPCR. Primers for promoter/exon amplification and cloning after bisulfite treatment. (0.04 MB DOC) [file pgen.1001203.s006.doc]

**SI Materials and Methods**

**Generation of the *Sp3* knockin homologous recombination construct**

A targeting vector containing the Sp3 E_553_D mutation was constructed as follows. The SUMO motif of mouse Sp3 is encoded by exon 5. A genomic 2.8 kb fragment containing exon 5 was obtained from a mouse cosmid clone by PCR amplification using intron-specific primers with overhanging NotI (5´-ACTGTGGCGGCCGCGAGTGCTGGGATTAAAGGTGTG-3´) and XhoI sites (5´-GTGTACCTCGAGAGTGAGTTCCAAGACAGCCAGG-3´), and cloned into NotI/XhoI-restricted *pBSIIKS* plasmid leading to *pBS-ki5*. Mutations that replaced the GAG codon to GAT (E_553_D) and in addition introduced silent codon mutations for genotyping by restriction analysis (GATATC; EcoRV) and PCR

(5´-TATCTTTAGATATCAGGATCAAGGAG**GAT**GAGCCTGACCCTGAAG-3´; underlined nucleotides differ from the wild type sequence) were introduced into *pBS-ki5* using the QuikChange XL site-directed mutagenesis kit (Stratagene).

As starting plasmid for the targeting vector we chose the *pPTloxPneo* plasmid [1] that contains a *PGK-hsv-tk* and a floxed *PGK-neo* cassette. A 2.5 kb *Sp3* gene fragment of intron 4 was cloned upstream of the floxed *PGK-neo* cassette (BamHI/KpnI restricted *pPTloxPneo*). The fragment was obtained by PCR using 129/ola mouse DNA as template and intron-specific primers with flanking BamHI (5´-ACTGTGGGATCCCTGTAGACCAGGCTGGCCTCATT-3´) and KpnI (5´-GTGTCAGGTACCCTCGGGAAAGAGTCAATGCTCTT-3´) sites. The *PGK-neo* cassette was subsequently replaced by a promoterless *IRES-lacZ-neo* cassette obtained as a 6.8 kb SalI fragment form *pGT1.8Iresßgeo* [2]. Finally, the mutagenized exon 5 containing fragment was introduced upstream of the *IRES-lacZ-neo* cassette via NotI and XhoI restriction.

**Genotyping of targeted Sp3 knockin mice by PCR**

DNA was prepared from tail snips and analyzed for the presence of the *Sp3wt* and the targeted *Sp3ki* allele by PCR. Three primers were used: (1) a sense primer directed to exon 5 of the *Sp3wt* allele (5´-atattaggatcaaggaagag-3´); (2) a sense primer directed to the mutant allele (5´-caggatcaaggaggatgag-3´); (3) an antisense primer directed to intron 5 of the *Sp3* gene (5´-gcgaatttctgaatgagg-´3). PCR conditions were 94°C for 1 min; 56°C for 1 min and 72°C for 1 min for 30 cycles.

**Transfection of ES cells and generation of chimeric and Sp3 SUMOylation- deficient mice**

E14 ES cells were electroporated with 15 μg of the NotI-linearized targeting vector. Clones were selected with G418 (200 μg/ml) and homologous recombination was analyzed by Southern blotting after BglII restriction of genomic DNA. A single clone out of >200 analyzed showed the expected DNA fragment. Additional Southern blots with EcoRV- and HpaI-restricted DNA and the probes indicated in Supplemental Figure 1A confirmed homologous recombination. The targeted ES cell clone was karyotyped and microinjected in C57BL/6 host blastocysts. Chimeric males were mated to C57BL/6 females. The F1 offspring harboring germline transmission of the targeted allele were bred to C57bl/6 mice to expand the stocks. The *IRES-lacZ-neo* cassette was removed by crossing heterozygous *Sp3wt/neo* mice with Cre recombinase expressing *CAG-Cre* mice [3]. Heterozygous offspring harboring the Sp3-SUMOylation-deficient *Sp3* (*Sp3ki*) allele were further expanded by crossing with C57bl/6 mice. *Sp3ki/ki* mice were obtained by intercrossing of *Sp3wt/ki* mice.

**Primers for RT-qPCR**

***Dmc1-1***

Forward: 5´-CCCAGATGTTGTCACGACTC-3´

Reverse: 5´-AGCGGTTATTGCGAAGGTGG-3´

***Dmc1-2***

Forward-2: 5´-TATCACTACTGGGAGCCAGG-3´

Reverse-2: 5´-AAGGCGATCTGGACGGAAAG-3

***Paqr6:***

Forward: 5´-TCCTTCGAGTCCATCAGGTC-3´

Reverse: 5´-TAAAGGTGTGCGCACAGCAG-3´

***Dnahc8:***

Forward: 5´-GGCCTAACGTGTTCTGGATG-3´

Reverse: 5´-GGGTGGATTCTGTGAGCTTC-3´

***Robo3:***

Forward: 5´-CTACAGAAGGGAGCACAGTC-3´

Reverse: 5´-ATGGCTCAGGAAGCTTGGTC-3´

***Rims3:***

Forward: 5´-CCCAAACCAGGCTCCAAATC-3´

Reverse: 5´-AGCACTTGTGGTCCATACGG-3´

***Vill:***

Forward: 5´-AACTGGGCTGTGTCCAGTAC-3´

Reverse: 5´-CCCCTTTGTTGCCTGCATTC-3´

***Smc1ß:***

Forward: 5´-ACACAGTTTTCGGCCTGCTC-3´

Reverse: 5´-CCAGAGTCAACACATGGCTG-3´

***Stag3:***

Forward: 5´-AGAGTCTGACGGCCAAAGAA-3´

Reverse: 5´-GCTTCTGGCAAAGTCCACTC-3´

***Sp1:***

Forward: 5´-GTTGGTGGCAATAATGGGGG-3´

Reverse: 5´-CCTGGGAGTTGTTGCTGTTC-3´

***Gapdh:***

Forward: 5´-AGACGGCCGCATCTTCTTGT-3´

Reverse: 5´-GCCTTGACTGTGCCGTTGAA-3´

**Primers for ChIP-qPCR**

***Dmc1* promoter:**

Forward: 5´-TCTCTAGCTAACCTCGAGCC-3´

Reverse: 5´-CCAGAATTCTAACGCCGACC-3´

***Paqr6* promoter:**

Forward: 5´-CCCCTAGATCCTAGAAAGAC-3´

Reverse: 5´-CTCAGCCCAGACCTGTTTG-3´

***Rims3* promoter:**

Forward: 5´-CACTTCTTCCAGGCCTACTC-3´

Reverse: 5´-TGCAAAGCCCAAGCCACGG-3´

***M4* promoter:**

Forward: 5´-TTTCCAAGAAGTCAGCCGGC-3´

Reverse: 5´-ACAGTACTAGGCTAAGGCCC-3´

***Unspecific region*:**

Forward: 5´-CTGCGTCTTTATGCTGCTGC-3´

Reverse: 5´-CCCAGTGTTAGCTTTGTGCC-3´

**Primers for promoter/exon amplification and cloning after bisulfite treatment**

***Dmc1 promoter (1):***

Forward-4: 5´-gtcacggtaccaTTTTTTTTTGTGTTATATTTAT-3´

Reverse-3: 5´-agtcaggatcccAAACAAACCCCCTTAATAAA-3´

***Dmc1 promoter/exon1 (2):***

Forward-2: 5´- gtcacggtaccaTTATTAAGGGGGTTTGTTT-3´

Reverse-2: 5´- agtcaggatcccAATTTTCCTCAAATTTTTCC-3´

***Paqr6 promoter:***

Forward: 5´- catgcggtaccgAGATTTTAGAAAGATTTTAGAATGG-3´

Reverse: 5´- agtcaggatccgTTATACCCTTAAAAATACTCAAAAC-3´

***Rims3 promoter (1):***

Forward-3: 5´-gtcacggtaccaGATATATTTAGAAGATTTAG-3´

Reverse-3: 5´-agtcaggatcctAATACAAAACCCAAACCA-3´

***Rims3 exon1 (2):***

Forward-2: 5´-gtcacggtaccaTGGTTTGGGTTTTGTATT-3´

Reverse-2: 5´-agtcaggatcctCAAAACCTACTAACTTAC-3´

**References**

1. Shalaby F, Rossant J, Yamaguchi TP, Gertsenstein M, Wu XF, et al. (1995) Failure of blood-island formation and vasculogenesis in Flk-1-deficient mice. Nature 376: 62-66.

2. Mountford P, Zevnik B, Duwel A, Nichols J, Li M, et al. (1994) Dicistronic targeting constructs: reporters and modifiers of mammalian gene expression. Proc Natl Acad Sci USA 91: 4303-4307.

3. Sakai K, Miyazaki J (1997) A transgenic mouse line that retains Cre recombinase activity in mature oocytes irrespective of the cre transgene transmission. Biochem Biophys Res Commun 237: 318-324.
